# Postoperative urinary retention after pelvic organ prolapse surgery: influence of peri-operative factors and trial of void protocol

**DOI:** 10.1186/s12905-021-01330-4

**Published:** 2021-05-11

**Authors:** B. C. Anglim, K. Ramage, E. Sandwith, E. A. Brennand, for the Calgary Women’s Pelvic Health Research Group, Erin A. Brennand, Shunaha Kim-FineMagali, Magali Robert, Colin Birch, Magnus Murphy, Kaylee Ramage, Emily Sandwith

**Affiliations:** Section of Female Pelvic Medicine and Reconstructive Surgery, Department of Obstetrics and Gynaeacology, Foothills Medical Centre, School of Medicine, University of Calgary, 1403 29 Street Northwest, Calgary, AB T2N 2T9 Canada

**Keywords:** Voiding dysfunction, Urinary retention, Postoperative voiding trial, Postoperative urinary retention

## Abstract

**Purpose:**

Transient postoperative urinary retention (POUR) is common after pelvic floor surgery. We aimed to determine the association between peri-operative variables and POUR and to determine the number of voids required for post-void residuals (PVRs) to normalize postoperatively.

**Methods:**

We conducted a retrospective cohort study of 992 patients undergoing pelvic floor surgery at a tertiary referral centre from January 2015 to October 2017. Variables assessed included: age, BMI, ASA score, anaesthesia type, type of surgery, length of postoperative stay, surgeon, bladder protocol used, and number of PVRs required to “pass” the protocol.

**Results:**

Significant risk factors for POUR included: placement of MUS during POP surgery, anterior repair and hysterectomy with concomitant sacrospinous vault suspension. A total of 25.1% were discharged requiring catheterization. Patients receiving a concomitant mid-urethral sling (MUS) were 2.2 (95% CI1.6–2.9) and 2.3 (95% CI 1.8–3.1) times more likely to have elevated PVR after their second TOV and third TOV (*p* < 0.0001), respectively, compared with those without concomitant MUS. Permitting a third TOV allowed an additional 10% of women to pass the voiding protocol before discharge. The median number of voids to pass protocol was 2. An ASA > 2 and placement of MUS were associated with increasing number of voids needed to pass protocol.

**Conclusions:**

While many women passed protocol by the second void, using the 3rd void as a cut point to determine success would result in fewer women requiring catheterization after discharge. Prior to pelvic floor surgery, women should be counselled regarding POUR probability to allow for management of postoperative expectations.

## Introduction

Transient postoperative urinary retention (POUR) is common following pelvic floor surgery surgery and occurs in 15–45% of women [1–3]. When POUR is not identified, it can lead to significant morbidity including prolonged bladder distension with associated detrusor injury, renal dysfunction secondary to ureteric reflux and urinary tract infections [4–6]. Undiagnosed POUR may also lead to distressing, emergency presentations to the emergency department for catherization after post-operative discharge.

Women are at higher risk of POUR following pelvic floor reconstructive surgery. This is likely due to tissue edema, changes in the urethrovesical junction and haematoma formation [7]. Small peripheral nerve endings required for bladder sensation can become temporarily disrupted, resulting in a transient neuropathy and resultant bladder dysfunction [4]. Previously evaluated risk factors for POUR include: lower body mass index (BMI), advanced age, higher stage of prolapse, anterior colporrhaphy, previous incontinence surgery, and high preoperative post void residuals (PVR) [8–12]. Intravenous fluid administration of > 750 mL or a bladder volume of ≥ 270 mL in the postanaesthetic recovery area has also been shown to increase the risk of POUR [13]. In addition to this, increased opioid administration is associated with a 1.5 times higher risk of developing POUR (OR 1.3) [14]. Interestingly, a study by Bracken et al. [15] showed that vaginal bupivacaine used at the time of midurethral sling (MUS) placement increased the rate of POUR but did not reduce pain levels or pain medication use [15].

The optimal mode of bladder filling prior to postoperative trial of void (TOV) is unknown. Two main methods of trial of void exist: (1) retrograde filling the bladder using the foley catheter left in situ, and (2) spontaneous bladder filling (16–20). Retrograde voiding trials have a sensitivity of 94.4% and a specificity of 58.1% in detection of urinary retention [16]. Spontaneous fill has a sensitivity of 100% and specificity of 25.8%; however, this method may take longer to complete than the retrograde approach due to the time needed for the bladder to naturally and passively fill [16]. A study by Pulvino et al. [17] compared retrograde fill of 300 ml using a foley catheter and spontaneous fill to determine TOV success. The retrograde fill technique resulted in statistically significantly more complete bladder emptying compared to the spontaneous fill. It also showed less heterogeneity in bladder volume during the TOV and less overdistension to bladder volumes over 450 ml [17]. Another study by Dolgun et al. [18] showed TOV success to be equal between spontaneous fill and retrograde groups. The spontaneous void group required women to void ≥ 150 ml to pass, compared to the retrograde fill group who had more stringent criteria and were required to void 200 ml and have a PVR < 100 ml. A similar percentage of women in both groups returned with urinary retention requiring catheter insertion [18].

POUR is a common occurrence following pelvic floor surgery and can lead to significant anxiety and distress if women are discharged with a catheter or self-catheterizing. The primary aim of this study was to determine which peri-operative factors were associated with developing POUR. This information was felt to be valuable for pre-operative patient counselling regarding expectations for postoperative catheterization. Our secondary aim was to determine the average number of voids required for post void residuals (PVRs) to normalize postoperatively.

## Materials and methods

We conducted a retrospective cohort study of patients who underwent pelvic floor reconstructive surgery with four surgeons at a tertiary referral centre from January 2015 to October 2017. This project was reviewed and approved by the Foothills Medical Centre Research Ethics Boards (IDs# CHREB150706). Surgeries included for analysis were obliterative procedures (with or without MUS) and reconstructive procedures that addressed the vaginal apex (including vaginal hysterectomy with sacrospinous or uterosacral vault suspension, with or without anterior and posterior colporrhaphy, sacrospinous vault suspension, sacrocolpopexy (SCP), with or without MUS). Cases were identified in the Section of Pelvic Medicine & Reconstructive Surgery Surgical Booking Database. Peri-operative data was entered as part of routine clinical practice in the city-wide inpatient EMR (Sunrise Clinical Manager) and abstracted by a FPMRS/Urogynecology fellow to a database designed for research purposes.

Single site prolapse surgeries such as isolated anterior or posterior repairs were not captured by the dataset. All MUS procedures at time of pelvic organ prolapse (POP) surgery were performed with the use of vaginal xylocaine 1% with 1:100,000 Epinephrine mixed 1:1 with sterile water, in the range of 10–40 ml for hydro dissection during placement. Patients were excluded from our study if they underwent hysterectomies that did not include an apical suspension procedure (such as those performed for non-prolapse indications), isolated incontinence surgeries, vesicovaginal and rectovaginal fistula repairs, as well as day surgery cases (such as dilation & curettage, laparoscopic resection of endometriosis). Patients who had pre-operatively elevated post-void residuals ≥ 150 ml were excluded from this dataset. Data were extracted from the patient’s post-operative electronic chart. Variables extracted included: age, body mass index (BMI), American Society of Anesthesiologists (ASA) score as a marker of health, previous pelvic floor surgery and type of procedure performed. Perioperative variables extracted included: length of hospital stay (in days), number of voiding trials in hospital and whether the patient was sent home with a catheter or self-catheterizing.

At the tertiary centre where the study was conducted, two bladder protocols are administered in the post-operative period at the attending surgeon’s discretion. The first (Retrograde Protocol) is carried out by retro-filling the bladder with 300 ml of normal saline or sterile water through the foley catheter which was left in situ overnight. The voided volume is measured in a voiding hat by nurses and post void residual (PVR) is determined based on the volume voided. The second and subsequent (Spontaneous Filling Protocol) bladder protocols involve removing the foley catheter and allowing the bladder to spontaneous fill. The patient must void within 4 h of catheter removal, and PVR is measured by bedside bladder scanner. Voided volumes are measured in a voiding hat by nurses and the PVR measured with a bladder scanner. If patients have two consecutive voids > 200 ml with a PVR ≤ 150 ml, then they are considered to have passed the bladder protocol and monitoring of voiding stops. If the first TOV is failed, then two further consecutive voids must be “passed” in order to pass the voiding protocol. If the PVR is > 250 ml, an in & out catheter is placed to both confirm PVR and decompress the bladder. With both of these protocols, the post-operative indwelling catheter is removed at 6 AM on Day 1 in compliance with Early Recovery After Surgery (ERAS) guidelines for minimal duration of catheterization [19]. Patients must pass a minimum of two consecutive TOVs in order to pass the voiding protocol. This is based on the unreliability of PVR measurements a the need for repetition to confirm consistency [20]. A study by Dunsmuir et. al., showed that only one-third of patients had approximately constant PVRs (variation in range < 120 mL), and so repeated transabdominal bladder ultrasound is required [21]. A patient may have a falsely low PVR and may represent with urinary retention if only one PVR is done as part of a TOV. Type of bladder protocol administered and results are then documented in the electronic patient chart.

If the patient has met all other criteria for discharge, but have not passed their TOV, they are given the option to perform self-catheterization after each measured void at home or to be discharged with an indwelling foley catheter. Those patients who elect to self-catheterize upon discharge can discontinue self- catheterization once their voiding pattern demonstrates voids of > 200 ml with a PVR ≤ 150 ml for 3 consecutive voids. Those who elect to discharge with a catheter are reviewed in clinic for a retrograde TOV performed 4–7 days after discharge. This is in keeping with a prospective randomized controlled trial by Schachar et al. which showed that women with POUR after prolapse surgery had a sevenfold higher risk of failed repeat office TOV if performed on postoperative day 4 compared to postoperative day 7 [22].

Statistics were conducted using Stata 16 (College Station, Texas). Descriptive statistics were used to describe the study sample, calculating proportions, mean, and median values for demographic characteristics. Elevated PVR was defined as having a PVR of greater than 150 cc. We calculated descriptive statistics, chi-square tests, and crude odds ratios for elevated PVR on the second void, stratifying the results by POP procedure type (reconstructive vs. obliterative) and presence of MUS procedure. Reconstructive procedures were then further stratified by concomitant versus previous hysterectomy to explore for effect of hysterectomy on POUR risk factors.

Sample size for regression-based analyses is difficult to compute a-priori. However, a general rule of 10 cases/events per regression variable is accepted for logistic regression. Our original model included over 10 variables and multiple interaction terms. For this reason, we estimated we would need 200 events for our logistic regression model. Recognizing that some cases may have missing information due to charting errors, we increased our sample size by 20% to 240 events. Estimating the prevalence of post-operative urinary retention to be 40% this would be a sample size of 600 women. However, recognizing that other forms of regression analysis would require samples larger than logistic regression due to multiple group comparisons, the same size was again increased by 50% to 900. Based on our average surgical volume, it was estimated that a review of all cases over 34 months would provide this volume (January 2015-October 2017).

We conducted several regression analyses, recognizing that the concept of POUR can be defined multiple ways. Binary logistic regression evaluated for the effect of patient age, BMI, ASA Score, bladder protocol type, anesthesia type (general vs regional), surgeon, concomitant hysterectomy, anterior and posterior vaginal wall repairs on the outcome of passing the TOV at the second post-operative void. Age and BMI were explored in linear and non-linear fashion. Interaction terms between age and BMI and combinations of surgical procedures were explored. We also explored the potential effect of surgeon on the voiding outcomes through mixed effects logistic regression and the value of the variance reported by McFadden’s R-squared (not reported).

Association with the absolute number of voids taken to pass the bladder protocol and peri-operative variables were explored by zero-truncated Poisson regression where the dependent variable is an observed, non-zero count, assumed to follow a Poisson distribution. The Poisson regression modelling examined whether any measured clinical variable (including age, BMI, MUS, ASA score, anaesthesia type, hysterectomy, uterosacral or sacrospinous suspension, and laparoscopic SCP) predicted the number of voids to pass the bladder protocol. Both crude odds ratios and adjusted odds ratios were calculated.

Plotting a histogram of the number of voids required to pass the TOV allowed us to determine where natural clustering of the data occurred, and to classify pattern groups of the number of voids needed to pass the TOV were performed which were ordinal in nature. We assessed the proportional odds assumption using the Brant test. Results of the Brant test indicated that the proportional odds assumption was not violated and that we can assume that the relationship between each pair of outcome groups is the same (i.e., being in class 2 or 3 compared to 1 is the same as being in group 3 compared to 2 and 1). We calculated both crude models (using the single predictor variable and the outcome of void class) and adjusted models (using all predictor variables and the outcome of void class).

For all of the regression modelling, we assessed for significance at the *p* < 0.05 level. For the binary outcome of passing TOV on the second void, sensitivity analyses using a cutpoint of a 3rd void was also performed. Use of multiple regression methodologies acted as sensitivity analysis to ensure our results were consistent across different ways of classifying POUR.

## Results

Overall, our study examined the association with procedure and demographic characteristics and POUR for 992 women receiving pelvic floor reconstructive surgery at a tertiary care centre. Demographic characteristics of our study sample are described in Table 1. A total of 25.1% were discharged home with an indwelling catheter or performing self-catheterization.

**Table 1:** Demographic characteristics of women undergoing pelvic floor reconstructive surgery from Jan 2015 to October 2017 (n = 992)

	Median (IQR)	Mean 95% CI	
Age (years)	62 IQR: 53–71	61.5 95% CI 60.7–62.3	
BMI	27.4 IQR: 24.3–31.2	28.4 95%CI 28.0–28.7	
Length of stay (days)	1 IQR: 1–2	2.0 95%CI 1.3–2.7	
Number of voids to pass bladder protocol	2 IQR: 2–3	3.0 95%CI 2.9–3.2	

		N	Proportion (%) 95% CI
ASA score (%)	1	420	42.5% 95%CI 39.4–45.6%
	2	505	51.1% 95%CI 47.9–54.2%
	3	63	6.4% 95%CI 5.0–8.1%
	4	1	0.1% 95%CI 0.01–0.7%
Anaesthesia type	Regional	89	9.0% 95%CI 7.4–10.9%
	General	902	91.0% 95%CI 89.1–92.6%
Surgery type	Colpocleisis	51	5.1% 95%CI 3.9–6.7%
	Sacrospinous Suspension	380	38.3% 95%CI 35.3–41.4%
	Uterosacral Suspension	261	26.3% 95%CI 23.7–29.1%
	Anterior Repair	587	59.2% 95%CI 56.1–62.2%
	Posterior Repair	715	72.1% 95%CI 69.2–74.8%
	Lap SCP	107	10.8% 95%CI 9.0–12.9%
	TVT	150	15.1% 95%CI 13.0–17.5%
	TOT	85	8.6% 95%CI 7.0–10.5%
	MUS	235	23.7% 95%CI 21.1–26.4%
	Vaginal hysterectomy	382	38.5% 95%CI 35.5–41.6%
Type of bladder protocol	Spontaneous filling	399	41.3 95%CI 38.2–44.4%
	Retrograde filling	568	58.7% 95%CI 55.6–61.8%
Voids to pass categories	1–3	573	75.1% 95%CI 71.9–78.0%
	4–8	130	17.0% 95%CI 14.5–19.9%
	9 or more	60	7.9% 95%CI 6.2–10.0%
Patients electing discharge with catheter	249	25.1% 95%CI 22.5–27.9%	

^*^Some numbers may not add up to 100% due to missing data

We examined characteristics of women having a PVR > 150 ml by obliterative vs. reconstructive procedures (Table 2). Overall, 51.2% (95%CI 48.0–54.3%) of women in our study had elevated PVR after their second void and 40.8% (95%CI 37.7–43.9%) had elevated PVR after their third void. Overall, those receiving a concomitant MUS procedure were 2.2 (95% CI 1.6–2.9) times as likely to have elevated PVR after their second TOV (*p* < 0.0001) and 2.3 (95% CI 1.8–3.1) times as likely to have elevated PVR after their third TOV (*p* < 0.0001). Elevated PVR after second and third void significantly differed by the presence of a MUS in reconstructive procedures, however, odds of elevated PVR were not significantly different by the presence of a concomitant MUS procedure for obliterative procedures, likely due to the relatively small sample size of women receiving both obliterative and MUS surgery. For obliterative procedures, no variable was associated with the odds of failing the bladder protocol, making pre-operative prediction of outcomes difficult in this group.

**Table 2:** Analysis of demographic characteristics and bladder outcomes of women undergoing pelvic floor reconstructive surgery, stratified by reconstructive vs obliterative procedures and presence of mid-urethral sling procedures (n = 992)

		All POP procedures				Obliterative procedures				Reconstructive procedures			
		Total	With MUS N (%)	Without MUS N (%)	*p* value	Total	With MUS N (%)	Without MUS N (%)	*p* value	Total	With MUS N (%)	Without MUS N (%)	*p* value
Total		992	235 (23.7%, 21.1–26.4%)	757 (76.3%, 73.6–78.9%)		51	4 (7.8%, 2.9–19.5%)	47 (92.2%, 80.5–97.1%)	**0.006**	941	231 (24.5%, 21.9–27.4%)	710 (75.5%, 72.6–78.1%)	*p* = 0.006
Age (years)	Mean (95%CI)	61.5 (60.7–62.3)	57.9 (56.3–59.5)	62.6 (61.8–63.5)	0.134	79.7 (77.9–81.4)	77.5 (73.3–81.7)	79.9 (78.0–81.8)	0.969	60.5 (59.8–61.3)	57.6 (56.0–59.2)	61.5 (60.6–62.4)	0.218
	Median (IQR)	62 (53–71)	57 (48–68)	64 (55–72)		80 (76–83)	77 (75.5–79.5)	80 (76–84)		62 (53–69)	57 (48–67)	63 (54–70)	
Length of stay (days)	Mean (95%CI)	2.0 (1.3–2.7)	1.8 (1.6–2.0)	2.1 (1.1–3.0)	0.078	1.6 (1.3–2.0)	2.0 (0.0–4.3)	1.6 (1.2–1.9)	0.208	2.0 (1.3–2.8)	1.8 (1.6–2.0)	2.1 (1.1–3.1)	0.077
	Median (IQR)	1 (1–2)	2 (1–2)	1 (1–2)		1 (1–2)	1.5 (1–3)	1 (1–2)		1 (1–2)	2 (1–2)	1 (1–2)	
Number of voids to pass bladder protocol	Mean (95%CI)	3.0 (2.9–3.2)	3.5 (3.1–4.0)	2.9 (2.7–3.1)	0.144	2.7 (2.2–3.2)	3.3 (0.0–7.2)	2.7 (2.2–3.1)	0.409	3.0 (2.9–3.2)	3.5 (3.0–4.0)	2.9 (2.7–3.1)	0.131
	Median (IQR)	2 (2–3)	2 (2–4)	2 (2–3)		2 (2–3)	2 (2–4.5)	2 (2–3)		2 (2–3)	2 (2–4)	2 (2–3)	
Patients who went home with a catheter	N	249	88	161	*p* < 0.0001	9	0	9	0.335	240	88	152	*p* < 0.0001
	Proportion (95%CI)	25.1% (22.5–27.9%)	37.4% (31.5–43.8%)	21.3% (18.5–24.4%)		17.6% (9.3–30.9%)	-	19.1% (10.1–33.3%)		25.5% (22.8–28.4%)	38.1% (32.1–44.5%)	21.4% (18.6–24.6%)	
Elevated PVR after second void	N	507	154	353	< 0.0001	24	1	23	0.357	484	153	331	0.0001
	Proportion (95%CI)	51.2% (48.0–54.3%)	65.5% (59.2–71.3%)	46.7% (43.2–50.3%)		47.1% (33.6–61.0%)	25% (0.08–92.9%)	48.9% (34.8–63.3%)		51.4% (48.2–55.7%)	66.2% (59.7–72.3%)	46.6% (42.9–50.4%)	
	Crude Odds Ratio (95%CI)	-	2.2 (1.6–2.9)	Reference	< 0.0001	-	0.3 (0.03–3.6)	Reference	0.375	-	2.25 (1.65–3.06)	Reference	< 0.0001
Elevated PVR after third void	N	404	132	272	< 0.0001	17	1	16	0.713	388	131	257	< 0.0001
	Proportion (95%CI)	40.8% (37.7–43.9%)	56.2 (49.8–62.4%)	36.0% (32.6–39.5%)		4.2% (2.6–6.7%)	25% (0.08–92.9%)	34.0% (21.7–49.0%)		95.8% (93.3–97.4%)	56.7% (50.1–63.2%)	36.2% (32.7 – 39.9%))	
	Crude Odds Ratio (95%CI)	-	2.3 (1.8–3.1)	Reference	< 0.0001	-	0.6 (0.06–6.7)	Reference	0.714		2.3 (1.7–3.1)	Reference	< 0.0001

*p value s reflect chi-square tests to determine if differences between those with and without MUS procedures are significant at the *p* < 0.05 level

We also examined the characteristics of women having a PVR > 150 ml by hysterectomy status, comparing those who had undergone previous hysterectomy and thus only had a vault suspension performed vs. those undergoing hysterectomy and concomitant apical suspension procedures. This stratification is shown in Table 3.

**Table 3:** Analysis of demographic characteristics and bladder outcomes of women undergoing pelvic floor reconstructive surgery, stratified by hysterectomy status

		Reconstructive surgery with previous hysterectomy				Reconstructive surgery with concomitant hysterectomy			
		Total	Sacrocolpopexy	Sacrospinous suspension	*p* value	Total	Uterosacral suspension	Sacrospinous suspension	*p* value
Total		308	105 (34.1%, 29.0–39.6%)	203 (65.9%, 60.4–71.0%)		364	228 (62.6%, 57.5–67.5%)	136 (37.4%, 32.5–42.5%)	
Age (years)	Mean (95%CI)	64.5 (63.3–65.7)	63.5 (61.8–65.3)	65.0 (63.5–66.6)	0.377	60.2 (59.1–61.4)	59.4 (57.9–60.9)	61.5 (59.9–63.2)	0.254
	Median (IQR)	65 (57.5–72)	63 (57–71)	66 (58–73)		62 (53–69)	61 (51–68)	62 (55–69)	
Length of stay (days)	Mean (95%CI)	2.8 (0.5–5.1)	1.8 (1.4–2.1)	3.3 (0.0–6.9)	0.622	1.8 (1.6–2.0)	1.7 (1.5–1.9)	1.8 (1.6–2.1)	0.524
	Median (IQR)	1 (1–2)	1 (1–2)	1 (1–2)		2 (1–2)	1 (1–2)	1 (1–2)	
Number of voids to pass bladder protocol	Mean (95%CI)	2.8 (2.5–3.0)	2.7 (2.4–3.0)	2.8 (2.4–3.1)	0.439	3.4 (3.1–3.7)	3.4 (3.0–3.9)	3.6 (3.1–4.1)	0.056
	Median (IQR)	2 (2–3)	2 (2–3)	2 (2–3)		2 (2–4)	2 (2–4)	3 (2–4)	
Patients who went home with a catheter	N	78	23	55	0.321	119	79	40	0.303
	Proportion (95%CI)	25.3% (20.8–30.5%)	21.9% (15.0–30.9%)	27.1% (21.4–33.6%)		32.7% (28.1–37.7%)	34.6% (28.7–41.1%)	29.4% (22.3–37.7%)	
Elevated PVR after second void	N	146	50	96	0.896	223	137	86	0.551
	Proportion (95%CI)	48.6% (42.0–53.2%)	48.1% (38.2–58.1%)	47.3% (40.3–54.4%)		61.3% (56.1–66.2%)	60.1% (53.4–66.5%)	63.2% (54.5–71.3%)	
	Crude Odds Ratio (95%CI)	-	1.0 (0.6–1.7)	Reference		-	0.9 (0.6–1.4)	Reference	
Elevated PVR after third void	N	116	39	77	0.941	189	120	69	0.726
	Proportion (95%CI)	37.8% (32.5–43.4%)	37.5% (28.2–47.5%)	37.9% (31.2–45.0%)		51.9% (46.8–57.0%)	52.6% (45.9–59.3%)	50.7% (42.0–59.4%)	
	Crude Odds Ratio (95%CI)	-	0.98 (0.6–1.6)	Reference		-	1.1 (0.7–1.7)	Reference	

The uterosacral suspension group for those with previous hysterectomy and the sacrocolpopexy for those with concomitant hysterectomy were excluded because of small sample size. Some values may not add to 100% due to missing values

In the reconstructive group *without* concomitant hysterectomy, logistic regression was used to examine the outcome of failing the bladder protocol after 2nd and 3rd TOV. Performance of a concomitant MUS was associated with odds of failing the bladder protocol on the 2nd TOV (aOR 3.08, 95%CI 1.67–5.68) and the 3rd TOV (aOR 2.96, 95%CI 1.65–5.33). Adjusted odds ratios (aORs) of failing the TOV after sacrospinous vault suspension and laparoscopic SCP in the absence of a hysterectomy were not significant (aOR 1.18, 95%CI 0.71–1.97 and aOR 1.02, 95%CI 0.621–1.69, respectively), nor was the crude OR comparing the two after 2nd and 3rd TOV, as shown in Table 3. For reconstructive procedures *with* a concomitant hysterectomy, MUS and anterior repair were significant predictors of failing the bladder protocol after the 2nd and 3rd TOV. When adjusting for MUS status and anterior repair, the adjusted odds ratios for sacrospinous vault suspension compared to uterosacral vault suspension were not significant (aOR 1.10, 95% CI 0.70–1.72 and aOR 0.87, 95%CI 0.56–1.36, respectively), nor were the crude ORs shown in Table 3.

In our crude logistic regression modelling, the OR of the Retrograde fill TOV compared to the spontaneous fill TOV was significant, with Retrograde TOV being 1.35 times (95%CI 1.04–1.75) as likely as natural fill TOV to fail the 2nd void and 1.45 times (95%CI 1.12–1.89) as likely to fail the 3rd void. However, in the adjusted model, these values were not significant for the 2nd void (aOR 1.16, 95%CI 0.89–1.52) or 3rd void (aOR 1.23, 95%CI 0.94–1.62), indicating that type of TOV does not influence the odds of passing the TOV on 2nd and 3rd TOV. The incidence rate ratio of this variable (comparing retrograde to spontaneous fill) in the adjusted zero-truncated Poisson regression model was 1.01 (95%CI 0.70–1.46), again suggesting it does not affect the absolute number of voids required to pass. This variable was then removed from the logistic regression modelling.

Three surgical procedures were found to be consistently associated with higher odds of having a PVR > 150 ml on the 2nd and 3rd postoperative voids (results not shown in table). These were: (1) performance of concomitant MUS procedure (after 2nd void: adjusted OR 2.22, 95%CI 1.62–3.05; and after 3rd void: adjusted OR 2.27; 95%CI 1.67–3.08); (2) anterior repair (after 2nd void: adjusted OR 1.55, 95%CI 1.18–2.06; and after 3rd void: adjusted OR 1.49, 95%CI 1.12–1.99); and (3) performance of hysterectomy (after 2nd void, adjusted OR 1.56, 95%CI 1.18–2.05 and after 3rd void, adjusted OR 1.71, 95%CI 1.29–2.25). Elevated BMI was found to be associated with declining odds of failing bladder protocol on 3rd TOV (adjusted OR 0.97, 95%CI 0.95–0.99), but not the 2nd TOV.

The findings of the mixed effects logistic regression indicated that the surgeon variable explained only 3.9% of the variance in the model predicting elevated PVR at the 2nd void and 7.5% of the variance in the model predicting elevated PVR at the 3rd void. This suggests the individual differences in each surgeon’s technique does not influence the outcome of POUR greatly.

In the adjusted Poisson regression model, we found that the presence of a MUS and having an ASA score of two or higher significantly increased the number of voids until the protocol was passed by 1.27 (95%CI 1.13–1.42) and 1.15 times (95%CI 1.03–1.27), respectively. Finally, although undergoing a vaginal hysterectomy or a sacrospinous suspension separately were not significantly associated with an increase in the number of voids until the bladder protocol was passed, having both a vaginal hysterectomy and sacrospinous suspension significantly increased the number of voids by 1.53 times (95%CI 1.18–1.97). Other predictors were not significantly associated with the number of voids until the protocol was passed (Table 4).

**Table 4:** Results of zero-truncated poisson regression analysis examining the association between clinical variables and number of voids to pass the bladder protocol

Clinical Variables	Crude Incidence rate ratio (RR, 95% CI)	*p* value	Adjusted Incidence rate ratio (RR, 95% CI)	*p* value
Age	99.9 (99.6–1.00)	0.664	99.9 (99.5–1.00)	0.984
BMI	1.00 (0.99–1.01)	0.944	99.7 (98.9–1.01)	0.470
**MUS**	**1.27 (1.14–1.41)**	** < 0.0001**	**1.27 (1.13–1.42)**	** < 0.0001**
**ASA score**	1.09 (0.995–1.20)	0.062	**1.15 (1.03–1.27)**	**0.010**
Anterior repair	**1.12 (1.02–1.23)**	**0.019**	1.05 (0.92–1.16)	0.451
Posterior repair	1.08 (0.97–1.20)	0.164	1.04 (0.93–1.17)	0.541
Anaesthesia type	1.15 (0.97–1.35)	0.100	1.13 (0.95–1.34)	0.177
Vaginal hysterectomy	1.03 (0.92–1.15)	0.656	0.80 (0.63–1.01)	0.064
Uterosacral suspension	**1.20 (1.09–1.33)**	** < 0.0001**	1.06 (0.78–1.44)	0.697
Sacrospinous suspension	1.07 (0.98–1.18)	0.139	0.94 (0.80–1.09)	0.400
Laparoscopic sacrocolpopexy	0.86 (0.73–1.00)	0.052	0.93 (0.77–1.12)	0.448
Obliterative	0.87 (0.70–1.08)	0.199	0.94 (0.73–1.22)	0.641
Vaginal hysterectomy and uterosacral suspension	**1.21 (1.09–1.35)**	** < 0.0001**	1.29 (0.90–1.86)	0.169
**Vaginal hysterectomy and sacrospinous suspension**	**1.28 (1.14–1.44)**	** < 0.0001**	**1.53 (1.18–1.97)**	**0.001**
Bladder protocol	1.02 (0.93–1.12)	0.663	0.95 (0.86–1.05)	0.284

Items in bold indicate significance at the *p* < 0.05 level

Using visual inspection of the data, we defined patterns of voids after surgery using natural breaks in the number of void passes after surgery. This defined three clusters patients requiring 1–3 voids, 4–8 voids, or > 8 voids to pass the protocol. Using ordinal regression (Table 5), we found that MUS, ASA score, and having a hysterectomy with sacrospinous suspension were significant predictors of void class. If a MUS was performed, the adjusted odds of taking longer (going up a void class) to void are 2.27 times higher (95%CI 1.52–3.40) assuming all other factors to be constant in the model. Having an ASA score of 2 or higher increased the odds of taking longer to void by 1.47 times (95%CI 1.01–2.14).

**Table 5:** Results of ordinal regression analysis examining the association between clinical variables and ordered void class

Clinical variables	Crude odds ratio (OR, 95% CI)	*p* value	Adjusted odds ratio (OR, 95% CI)	*p* value
Age	1.00 (0.98–1.01)	0.726	1.00 (0.98–1.01)	0.717
BMI	0.98 (0.95–1.01)	0.203	**0.97 (0.94–1.00)**	**0.047**
**MUS**	**2.21 (1.53–3.19)**	** < 0.0001**	**2.27 (1.52–3.40)**	** < 0.0001**
**ASA score**	1.19 (0.85–1.65)	0.314	**1.47 (1.01–2.14)**	**0.046**
Anterior REPAIR	**1.58 (1.13–2.22)**	**0.008**	1.47 (0.96–2.25)	0.074
Posterior repair	0.93 (0.65–1.34)	0.707	0.71 (0.48–1.06)	0.091
Vaginal hysterectomy	**2.13 (1.53–2.96)**	** < 0.0001**	1.17 (0.59–2.33)	0.6466
Uterosacral suspension	**1.75 (1.22–2.52)**	**0.002**	1.53 (0.83–2.82)	0.170
Sacrospinous suspension	1.33 (0.95–1.86)	0.092	0.90 (0.50–1.60)	0.715
Laparoscopic sacrocolpopexy	0.69 (0.40–1.21)	0.196	1.02 (0.51–2.04)	0.952
Obliterative	0.75 (0.35–1.58)	0.445	1.57 (0.61–4.00)	0.347
**Vaginal hysterectomy and sacrospinous suspension**	**2.34 (1.58–3.47)**	** < 0.0001**	2.18 (0.96–4.96)	0.062
Bladder protocol	1.26 (0.90–1.76)	0.175	1.01 (0.70–1.46)	0.942

^*^Crude ORs calculated using the outcome of void class

Items in bold indicate significance at the *p* < 0.05 level

## Discussion

This rigorous analysis of POUR following pelvic floor reconstructive surgery provides valuable information for clinicians which can help counsel women preoperatively and manage their postoperative expectations where postoperative catheterization may be required. In our analysis of all pelvic floor procedures, 51.2% passed the TOV after the 3rd TOV compared to 40.8% after the 2nd TOV, equating to an extra 10.4% passing the TOV and not requiring catheterization if a 3rd TOV was performed. This difference was similar when accounting for surgery with or without MUS.

Previous studies have shown that lower BMI, older women and anterior colporrhaphy are associated with higher risk of POUR [8–12]. In our analysis we found only modest differences in the reconstructive vs. obliterative approaches. This difference was primarily driven by reconstructive surgery with a hysterectomy. In the regression modelling, which includes all cases and thus does not lose power like a stratification approach, the effect of hysterectomy was only in the presence of a concomitant sacrospinous suspension. This may be due to longer operation times, higher intraoperative blood loss [3] or perhaps irritation to the pudendal nerves in the region of the sacrospinous suspension [23]. Interestingly, age and BMI were not strongly predictive in the regression models we used. In the truncated Poisson and ordinal regression models, an ASA score greater than 2 (a marker for medical co-morbidities) was associated with increasing number of voids needed to pass protocol. This suggests that rather than parameters such as age and BMI impacting ability to void after surgery, it is a woman’s overall level of health that is associated with POUR. We also did not find that a woman’s attending surgeon was predictive of the risk of experiencing POUR. The small variance contributed to the model by differing surgeons reassures that these associations are common to all four surgeons and not influenced by individual differences in technique.

In our study, placement of MUS was consistently the most significant risk factor for POUR. This is likely due to the fact that incontinence surgery aims to correct urethral hypermobility and are inherently designed to cause some degree of urethral obstruction [4]. Women who are fearful or unable to deal with elevated residuals after surgery (such as those experiencing issues with dexterity, obesity, or anxiety) may want to consider staging their incontinence procedure after POP procedure as concomitant sling at the time of POP surgery increased the risk of POUR [24].

Rates of failure of 2nd and 3rd TOVs are very high at our institution ranging from 36.1% (for POP procedures without MUS) to 56.2% (for POP procedures with MUS) after the 3rd TOV. This may be due to the early removal of catheter at 6am in keeping with ERAS guidelines. This would be in keeping with prior studies that suggest higher rates of successful TOV with longer catherization [25]. While early catheter removal is compliant with ERAS guidelines, it may result in significant proportions of women requiring intermittent catheterization for POUR and resultant increased UTIs. The possibility of being discharged home with an indwelling catheter or self-catheterizing can trigger anxiety and may also increase the risk of infection, which may impact patient satisfaction with their surgical experience [26]. This is of particular relevance for women who are scheduled for day case surgery and are keen not to be discharged with a catheter or self-catheterizing. Such information should be relayed to the day unit nursing staff and, where resources exist, an extra, third TOV should be attempted. Women at higher risk of POUR based on risk factors previously mentioned may benefit from being placed at the start of the day, thus benefiting from a longer time in PACU for their TOV. Future research could examine the impact of catheterization for POUR and its relationship to women’s perception of the surgery experience.

Finally, our study presents normative data on the number of voids required for PVRs to normalize postoperatively. Prior work suggests that on average 2–3 TOV are performed in the postoperative period, either on the ward or in the day care unit/post anesthetic care unit [15, 16]. Our study found the median number of voids to pass protocol was 2 for both reconstructive and obliterative procedures and the natural patterns of voiding identified in our analysis (1–3, 4–8, or > 8 voids to pass TOV) corresponds to the 75th percentile, 75th–95th percentile, and greater than the 95th percentile. This suggests that requiring > 3 voids to pass a bladder protocol should be the definition of POUR, and that patients requiring 8 or more voids are true outliers. Patients and the nurses who provide post-operative case to them should be taught that it is perfectly normal to require 3 voids for the post-void residual to normalize.

Strengths of our study include the large sample size (n = 992) and the level of peri-operative details available. The number and details of the voiding trials were accessed on the electronic surgical patient chart, which allowed a detailed analysis of the number of TOV and voiding parameters used. Limitations include not being able to account for intraoperative fluid administration in our model, as it has been suggested that volumes ≥ 750 ml increase risk of POUR [13, 14]. However, our centre adheres to ERAS principles including judicious use of peri-operative IV fluids and TOV did not start until post-operative day 1 meaning most intra-operative IV fluid would have been dealt with by diuresis overnight. While women with pre-existing PVR > 150 ml were not included in this cohort, information regarding other voiding parameters such as speed of urinary stream and shape of Uroflowmetry curve were not included to assess their value as predictive factors. A further limitation is that our results are not generalizable to patients being discharged on the day of surgery, where a higher number of patients have been shown to fail the voiding protocol and require catheterization [27]. Finally, it is possible that our sample size was underpowered to detect differences in some of the measured variables (e.g., differences between surgery types).

## Conclusion

POUR is common after pelvic floor surgery. A handful of POUR risk factors have been identified by our study, including MUS placement and concomitant hysterectomy with sacrospinous vault suspension. However, POUR still occurs in women without these risk factors making it difficult to predict pre-operatively. Normalization of PVR usually takes two voids, but a third void may be required. In women who are at higher risk of POUR and where day surgery is planned, surgeons should consider placing them at the start of the operative list to allow for time to void postoperatively. Women should be counselled regarding high rates of POUR in advance of surgery to allow them to manage postoperative expectations.

## Data Availability

The datasets used and/or analysed during the current study available from the corresponding author on reasonable request.
